# Correcting for unequal variance in signal detection models using response time

**DOI:** 10.1016/j.isci.2026.114998

**Published:** 2026-02-11

**Authors:** Kiyofumi Miyoshi, Dobromir Rahnev, Hakwan Lau

**Affiliations:** 1 Graduate School of Informatics, Kyoto University, Kyoto, Japan; 2 School of Psychology, Georgia Institute of Technology, Atlanta, GA, USA; 3 Center for Neuroscience Imaging Research, Institute for Basic Science, Suwon, South Korea; 4 Department of Biomedical Engineering, Sungkyunkwan University, Suwon, South Korea; 5 Department of Intelligent Precision Healthcare Convergence, Sungkyunkwan University, Suwon, South Korea

**Keywords:** Classification Description, Neuroscience, Behavioral neuroscience, Psychology

## Abstract

This study examines signal detection theory (SDT) analysis of perceptual detection performance using response time (RT) data. A defining feature of detection tasks is the asymmetry between trials with stimulus presence and absence, often reflected in asymmetric type-1 ROC curves. This asymmetry indicates greater signal variability in stimulus-present trials, which contradicts canonical assumptions in equal-variance SDT models. Across multiple datasets, we implemented an unequal-variance SDT model using RT data and compared it with the traditional confidence-based method. RT-based estimates of SDT parameters—*SD* ratio (σ) and mean difference (μ)—aligned closely with confidence-based estimates. The resulting sensitivity measure, *d*_*a*_—an unequal-variance extension of *d′*—derived from RT and confidence, showed strong consistency. Notably, conventional *d′* systematically overestimated detection performance compared to the *d*_*a*_ measures, highlighting the importance of accounting for unequal variance. RT-based SDT analysis offers a cost-effective alternative for robustly quantifying detection performance, particularly when confidence ratings are impractical.

## Highlights

• RT and confidence data were used for unequal-variance signal detection analysis
• Both measures produced similar *SD* ratio and mean difference estimates
• They yielded highly consistent estimates of *d*_*a*_ (unequal-variance extension of *d′*)
• Conventional *d′* systematically overestimated detection performance relative to *d*_*a*_

## Graphical abstract

**Figure:**
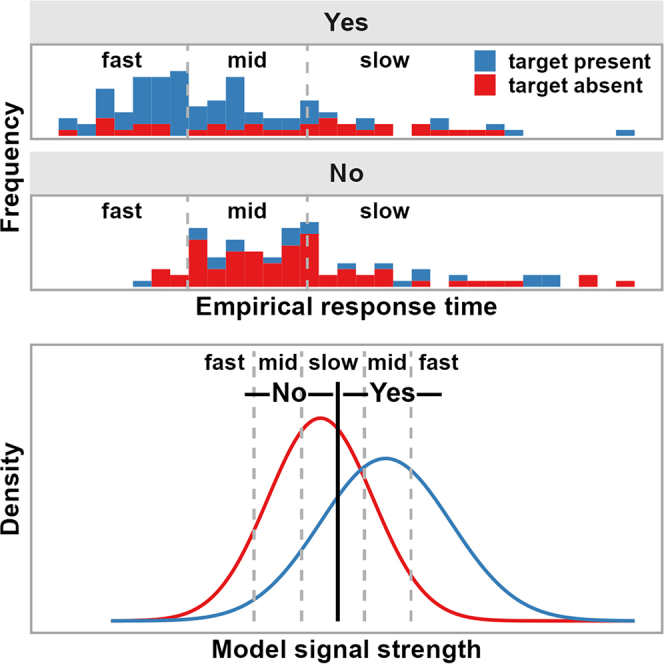

## Introduction

One of the central experimental paradigms in psychophysics is the yes/no detection task, in which observers report whether a target stimulus is present or absent (hereafter simply “detection”). This paradigm has long served as a primary method for probing the occurrence of subjective perception^1^—and continues to play a central role in multiple domains of research on perception.^2^^,^^3^^,^^4^^,^^5^

Analysis on detection performance typically focuses on the proportion of “yes” responses in target-present and target-absent trials—referred to as hits and false alarms (FAs). Applying the inverse of the cumulative Gaussian function (*z* transformation) to hit and FA rates, and taking the difference, yields *d′*, a sensitivity index based on the equal-variance signal detection theory (SDT) model.

(Equation 1) $$d^{\prime }=z\left(hit\;rate\right)-z\left(FA\;rate\right)$$

**Figure 1:**
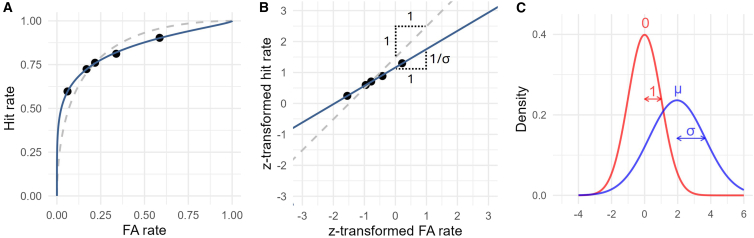
Example type-1 ROCs and the unequal-variance SDT model (A) From left to right, empirical type-1 ROC plots multiple pairs of hit and FA rates linked with progressively more lenient criteria (black dots). In detection tasks, empirical type-1 ROCs are often asymmetric, which can be captured by the unequal-variance SDT model, as illustrated by the solid navy curve. When a type-1 ROC is constructed with RT or confidence data, the midpoint (third point from the left in this example) is determined solely by yes/no responses. Conventional *d′* is calculated exclusively from this midpoint, assuming the equal-variance SDT model. This model predicts a symmetric ROC curve passing through the midpoint (dashed gray curve), which may misrepresent the observer’s actual performance. (B) The *z*ROC plot, in which hit and FA rates are *z*-transformed, is commonly used to visualize the internal *SD* ratio. In this space, SDT-based ROCs are linear, with their slope corresponding to the ratio of internal *SD*s. The equal-variance model, therefore, predicts a slope of 1, while the unequal-variance model predicts a slope of 1/σ (see panel c). (C) The unequal-variance SDT model captures type-1 ROC data through the σ and μ parameters, offering a more appropriate performance assessment via the *d*_*a*_ measure (see Equation 2). Due to the variance inequality, the target-present distribution (blue) shows substantially higher likelihood than the target-absent distribution (red) on the right side of the signal continuum. This is reflected in the steep rise of the type-1 ROC curve on the left—the slope of the model-based ROC at each point corresponds to the likelihood ratio between the two underlying distributions.

Although *d′* is one of the most popular metrics in psychophysics, its use in detection tasks demands caution.^6^^,^^7^^,^^8^^,^^9^ This is because target-present trials often exhibit greater internal signal variance than target-absent trials, as indicated by type-1 receiver operating characteristic (ROC) analysis (Figure 1; the term “type-1” refers to the classification of objectively defined external stimulus states, such as stimulus presence and absence in the present case).^10^^,^^11^^,^^12^ Specifically, while conventional *d′*, standing on the equal-variance SDT model, assumes symmetric ROCs, empirical ROC data in detection tasks are often asymmetric (Figure 1A), indicating the presence of unequal variance (Figure 1C).

Accordingly, *d*_*a*_—an unequal-variance extension of *d′*—is widely recommended for use in detection tasks and is defined as follows.

(Equation 2) $$d_{a}=\frac{\mu }{\sqrt{\frac{\left(1+\sigma^{2}\right)}{2}}}$$

The parameters μ and σ denote the mean and *SD* of the target-present distribution, respectively, and *d*_*a*_ quantifies the distance between the two internal distributions, standardized by their root-mean-square *SD* (Figure 1C). When σ = 1, *d*_*a*_ reduces to conventional *d′*.^13^

**Figure 2:**
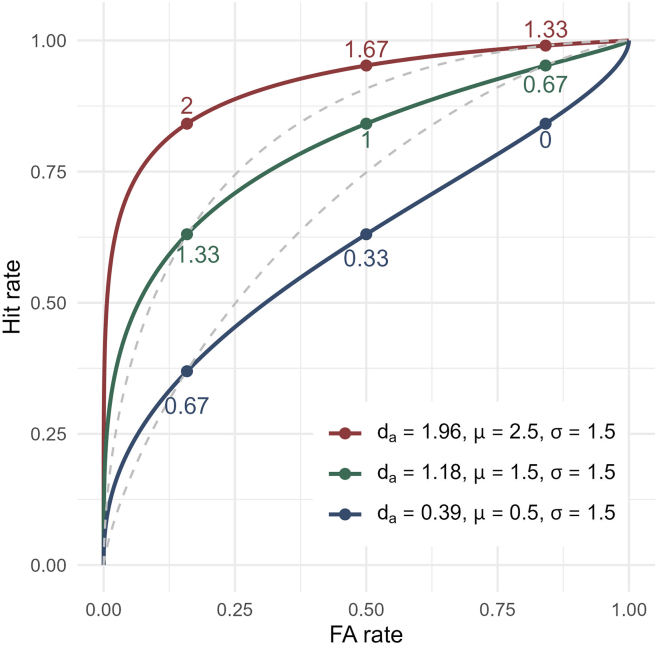
Criterion dependence of conventional *d′* Three iso-sensitivity curves are derived from the unequal-variance SDT model (σ = 1.5), with the μ parameter set to 0.5, 1.5, and 2.5 (corresponding to *d*_*a*_ = 0.39, 1.18, and 1.96). Response criteria of −1, 0, and 1 yield nine ROC points, with annotation values indicating the conventional *d′* calculated from those points. Compared with the true *d*_*a*_ values, conventional *d′* overestimates (or underestimates) detection performance when calculated from more conservative (or more lenient) data points. The dashed curves further illustrate this criterion dependence by showing equal-variance ROCs derived from two different points on the green unequal-variance ROC. The conservative point produced *d′* = 1.33, which overestimates the true sensitivity of 1.18, whereas the lenient point yielded *d′* = 0.67, which underestimates it.

Figure 2 illustrates how conventional *d′* misestimates detection sensitivity when the unequal-variance SDT model (σ = 1.5) is the truth data-generating process. Across three unequal-variance iso-sensitivity curves (*d*_*a*_ = 0.39, 1.18, 1.96), nine ROC points are plotted with corresponding conventional *d′* values derived using Equation 1. Critically, conventional *d′* overestimates sensitivity relative to ground-truth *d*_*a*_ with conservative response criteria (ROC points on the left) and underestimates it with liberal criteria (points on the right). This criterion-dependent misestimation arises from the asymmetry of detection ROCs under σ > 1, which exhibit greater expansion on the left.

This bias has serious implications for practical data analysis. When conventional *d′* is used to quantify detection sensitivity, individuals or conditions with conservative (or lenient) criteria can have their performance systematically overestimated (or underestimated). In Figure 2, the blue ROC even describes a scenario where estimated *d′* ranges from moderate to zero depending on criterion placement. This poses a critical problem for studies seeking to infer the occurrence of conscious perception by testing whether *d′* exceeds chance. Namely, although this example observer has above-chance detection sensitivity (ground-truth *d*_*a*_ = 0.39), applying the equal-variance SDT model can mistakenly indicate complete detection failure (*d′* = 0) when the criterion is sufficiently lenient.

Problems also arise when using *d′* to compare performance in detection tasks with performance in other paradigms, particularly “pattern-discrimination” tasks. These tasks typically involve the binary classification of distinct stimulation patterns—for example, “clockwise” vs. “counterclockwise” orientation, “upward” vs. “downward” motion, or “target on the left” vs. “target on the right” in a two-alternative forced-choice (2AFC) task.^14^^,^^15^^,^^16^^,^^17^^,^^18^^,^^19^ The equal-variance assumption underlying *d′* is known to be more tenable in pattern-discrimination tasks, where relative comparison between two stimulus categories effectively cancels out variance differences.^13^^,^^18^^,^^20^ Accordingly, failing to apply *d*_*a*_ instead of *d′* in detection tasks risks overestimating or underestimating performance relative to pattern-discrimination tasks.

Despite these caveats, *d′* remains in common use for evaluating detection performance, likely because internal variance inequality cannot be estimated from a single pair of hit and FA rates. Specifically, the fitting of the unequal-variance SDT model requires multiple pairs of hits and FAs—i.e., multiple points in type-1 ROC space (Figure 1)—typically obtained through base-rate manipulations, payoff manipulations, or the collection of confidence ratings (see method details).^13^ However, these methods (especially the first two) impose substantial cognitive and logistical costs, which can be especially prohibitive in studies involving children or non-human animals. Moreover, while methods for collecting confidence data from animals have been developed,^21^^,^^22^^,^^23^ their implementation often incurs the cost of additional behavioral training.

Therefore, this study investigated the use of response time (RT) to fit the unequal-variance SDT model as a cost-effective alternative to conventional methods. The key idea is that a faster RT is considered a stronger indication of the choice made.^24^ By applying progressively lenient RT cutoffs, multiple pairs of hits and FAs can be obtained, as visualized in type-1 ROC space (see Figure S1 for further details). While RT-based type-1 ROC analysis has been explored in earlier work, these studies mostly focused on model-free measures such as the area under the curve (AUC).^25^^,^^26^^,^^27^

The following presents unequal-variance SDT analyses based on both RT and confidence across multiple visual detection datasets. These analyses allow a systematic evaluation of the utility of RT-based analysis, which may offer a principled behavioral assessment while bypassing costly experimental setups.

## Results

**Figure 3:**
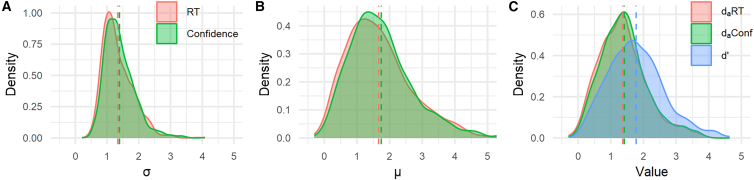
Estimated parameter distributions for visual detection tasks Data from 1,152 individuals across 11 datasets were aggregated for visualization (dashed lines indicate corresponding mean values). (A) The σ distributions derived from RT and confidence were highly consistent. (B) The μ distributions obtained from RT and confidence also showed strong consistency. (C) The *d*_*a*_ distributions derived from RT and confidence closely overlapped, while *d′*—which does not incorporate RT or confidence data—overestimated task performance compared to the *d*_*a*_ measures.

**Figure 4:**
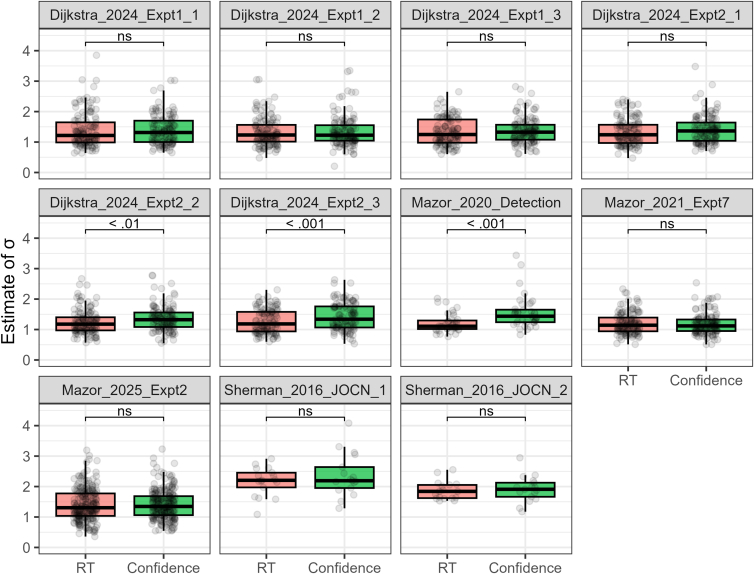
Estimates of σ for each dataset The estimates derived from RT and confidence were largely consistent. While significant differences between their mean values were observed in three datasets, the numerical difference across all datasets was only 6.3% (the across-dataset average of mean σ estimates was 1.43 for RT and 1.52 for confidence). Paired *t*-tests were used for statistical comparisons.

**Figure 5:**
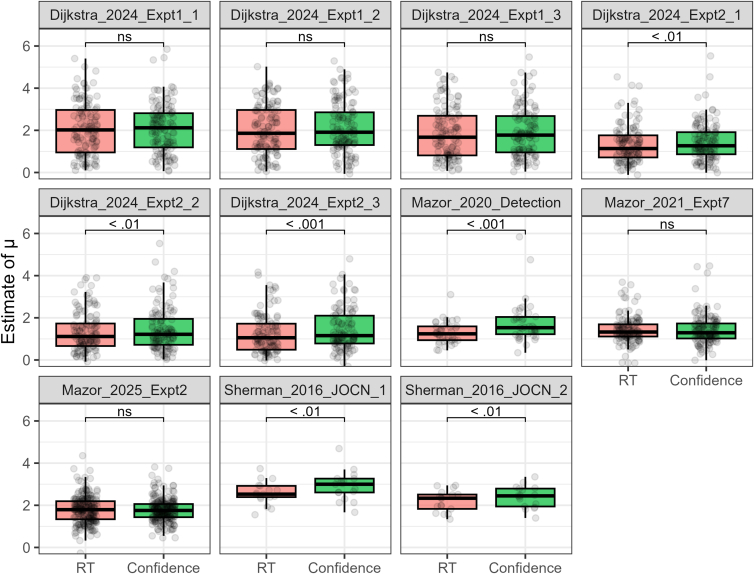
Estimates of μ for each dataset RT- and confidence-based estimates showed a high degree of consistency. Although significant differences between their mean values were observed in six datasets, the difference across all datasets was only 8.6% (the across-dataset average of mean μ estimates was 1.74 for RT and 1.89 for confidence). Paired *t*-tests were used for statistical comparisons.

**Figure 6:**
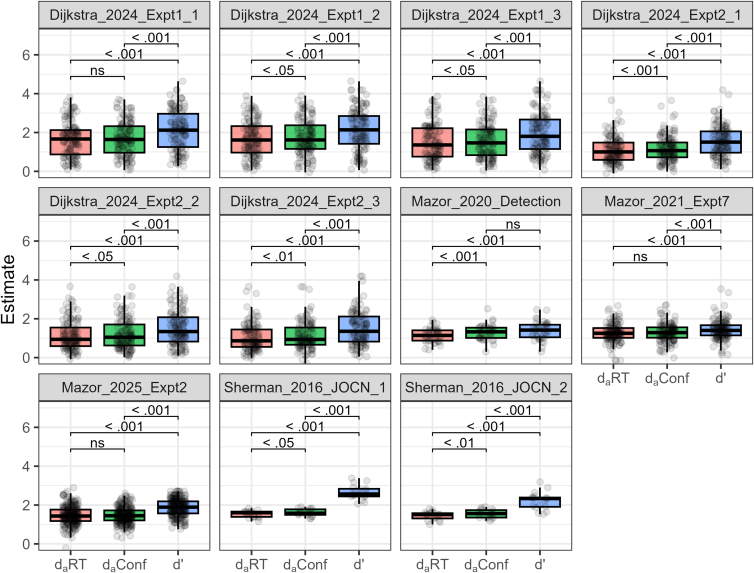
Sensitivity measures estimated for each dataset Conventional *d′* overestimated detection performance compared to *d*_*a*_ measures. The across-dataset average of mean *d′* values (1.86) was substantially higher than that of mean RT-based *d*_*a*_ values (1.36) and that of mean confidence-based *d*_*a*_ values (1.43). Paired *t*-tests were used for statistical comparisons (*p*-values uncorrected).

We selected 11 yes/no visual detection datasets^2^^,^^3^^,^^18^^,^^28^^,^^29^ (Table 1) and conducted unequal-variance SDT analyses (see method details). Figure 3 displays density plots of parameter estimates collapsed across all datasets, while Figures 4, 5, and 6 present separate plots for individual datasets. Importantly, estimates of σ—the *SD* ratio between target-present and target-absent trials—derived from both RT and confidence tended to exceed 1. The σ distributions based on RT and confidence were largely overlapping, with paired *t*-tests showing significant differences in their mean values for 3 of the 11 datasets (*p* < 0.05; Figure 4). Note that Sherman_2016_JOCN_1 and Sherman_2016_JOCN_2, which included trials of varying difficulty (via an online staircase procedure), yielded relatively large σ estimates. The average of the dataset means of σ was 1.43 (*SD* = 0.31) for RT and 1.52 (*SD* = 0.34) for confidence, corresponding to a difference of only 6.3%.

**Table 1:** Visual detection datasets

Dataset	Subjects	Trial/Sub	Confidence scale	Task	Specification
Dijkstra_2024_Expt1_1	125 (130)	96–192	Continuous (0–100), made into 3 levels	Grating detection	*ConditionID* = 1
Dijkstra_2024_Expt1_2	125 (130)	96–192	Continuous (0–100), made into 3 levels	Grating detection	*ConditionID* = 2
Dijkstra_2024_Expt1_3	120 (130)	96–192	Continuous (0–100), made into 3 levels	Grating detection	*ConditionID* = 3
Dijkstra_2024_Expt2_1	123 (127)	96	Continuous (0–100), made into 3 levels	Grating detection	*ConditionID* = 1
Dijkstra_2024_Expt2_2	123 (127)	96	Continuous (0–100), made into 3 levels	Grating detection	*ConditionID* = 2
Dijkstra_2024_Expt2_3	112 (127)	96	Continuous (0–100), made into 3 levels	Grating detection	*ConditionID* = 3
Mazor_2020_Detection	39 (46)	200–240	6 levels	Grating detection	*Condition* = "Detection"
Mazor_2021_Expt7	127 (136)	96	Continuous (0–1), made into 3 levels	Grating detection	
Mazor_2025_Expt2	224 (224)	32	Continuous (0–1), made into 3 levels	Letter detection	
Sherman_2016_JOCN_1	17 (18)	192–816	4 levels	Grating detection	*Condition* = 1
Sherman_2016_JOCN_2	17 (18)	192–816	4 levels	Grating detection	*Condition* = 2

The “Subjects” column lists the number of individuals included in the analysis; values in parentheses reflect the counts prior to data exclusion. The “Trial/Sub” column refers to the number of trials per individual. The “Specification” column details which experimental condition was selected from studies that included multiple conditions; variables shown in italics correspond to column headers in the source datasets.

Similarly, the μ distributions based on RT and confidence largely overlapped, with paired *t*-tests indicating significant differences in their mean values for 6 of the 11 datasets (*p* < 0.05; Figure 5). The average of the dataset means of μ was 1.74 (*SD* = 0.45) for RT and 1.89 (*SD* = 0.47) for confidence, amounting to an 8.6% difference.

The distributions of *d*_*a*_ based on RT and confidence overlapped substantially, with paired *t*-tests showing significant differences in their mean values for 8 of the 11 datasets (*p* < 0.05; Figure 6). As mentioned above, σ and μ estimates from RT were slightly smaller than those from confidence; however, this reduction affected both the numerator and denominator of Equation 2, leaving the resulting *d*_*a*_ largely unchanged. The average of the dataset means of *d*_*a*_ was 1.36 (*SD* = 0.23) for RT and 1.43 (*SD* = 0.22) for confidence, a difference of only 5.1%. In other words, although paired *t*-tests indicated statistical significance for several datasets, these results are primarily driven by very strong correlations between RT- and confidence-based *d*_*a*_ (shown later in Figure 9), with the actual numerical differences remaining minimal.

Importantly, *d′* derived solely from yes/no responses consistently overestimated detection performance relative to the *d*_*a*_ measures (Figure 3). Paired *t*-tests revealed that mean *d′* was significantly higher than mean RT-based *d*_*a*_ in all 11 datasets and significantly exceeded mean confidence-based *d*_*a*_ in 10 datasets (*p* < 0.05; Figure 6). The average of the dataset means of *d′* was 1.85 (*SD* = 0.41), which is 36% higher than that of RT-based *d*_*a*_ (*M* = 1.36, *SD* = 0.23) and 29% higher than that of confidence-based *d*_*a*_ (*M* = 1.43, *SD* = 0.22).

Moreover, as expected from Figure 2, the performance overestimation by *d′* relative to *d*_*a*_ was more pronounced for subjects with stricter response criteria. To quantify this, we estimated each subject’s criterion *c* using Equation 3 and correlated it with the difference between *d′* and *d*_*a*_ (i.e., performance misevaluation index).

(Equation 3) $$c=-\frac{z\left(hit\;rate\right)+z\left(FA\;rate\right)}{2}$$

Criterion *c* correlated significantly with the performance misevaluation index in all 11 datasets when *d*_*a*_ was derived from RT (*p* < 0.05, mean *r* = 0.53), and in 10 datasets when *d*_*a*_ was estimated from confidence (*p* < 0.05, mean *r* = 0.59).

These observations highlight a key implication: failing to account for unequal variance can lead to inaccurate estimates of visual detection performance, severely compromising comparisons across individuals or conditions associated with different response biases. Moreover, using *d′* may misrepresent detection performance relative to pattern-discrimination performance, as the equal-variance assumption is more reliably met in pattern-discrimination tasks, making the use of *d′* less of an issue.^13^^,^^18^^,^^20^ RT-based *d*_*a*_, which properly accounts for internal variance structure, offers a robust solution here, being less prone to response bias and providing better comparability across different experimental setups.

**Figure 7:**
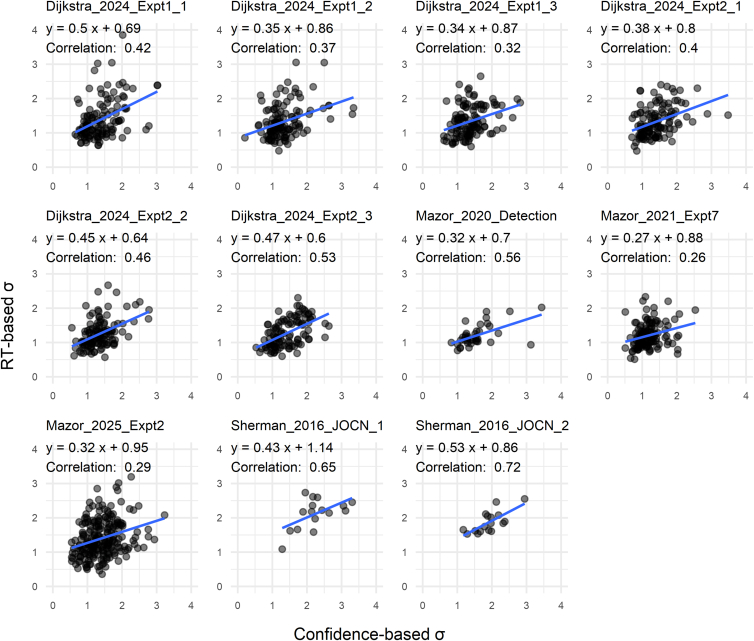
Correlation plots for the σ parameters Across datasets, σ estimates based on RT and confidence exhibited moderate correlations between individuals, suggesting that internal variance can be estimated with reasonable consistency based on RT data.

**Figure 8:**
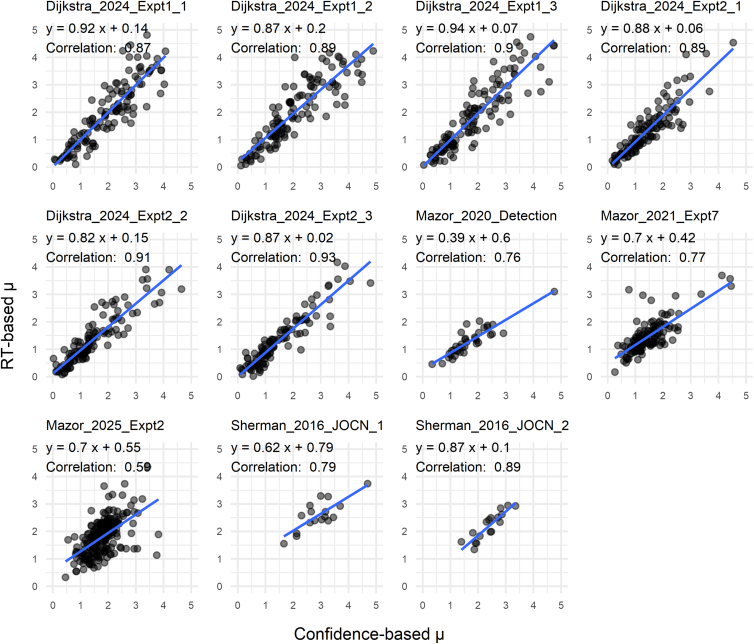
Correlation plots for the μ parameters Across datasets, μ estimates based on RT and confidence showed solid correlations between individuals. The strong correlations are expected, as μ estimates are strongly influenced by yes/no response data.

**Figure 9:**
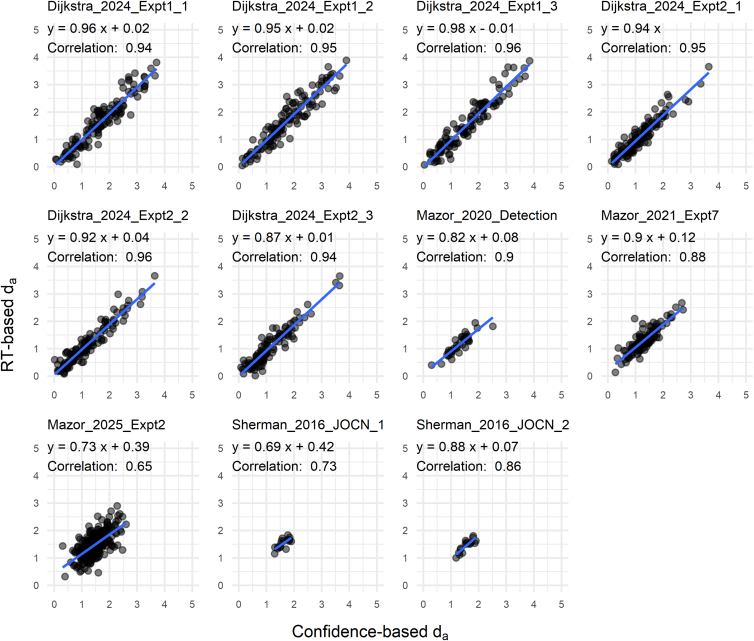
Correlation plots for *d*_*a*_ Across datasets, *d*_*a*_ estimates based on RT and confidence showed robust correlations between individuals, indicating that RT data provide a reliable basis for estimating *d*_*a*_.

We then examined across-subject correlations of parameter estimates derived from RT and confidence for each dataset (Figures 7, 8, and 9). Pearson’s correlations for σ were moderately high across all datasets (*p* < 0.05 for all; mean *r* = 0.45), indicating that RT-based estimation provides a viable means of capturing individual differences in internal variance inequality (Figure 7). Estimates of μ derived from RT and confidence were robustly correlated for each dataset (*p* < 0.05 for all datasets; mean *r* = 0.84; Figure 8). This is expected, as the μ parameter is strongly constrained by yes/no responses, which are shared between the two estimation approaches.

The *d*_*a*_ values estimated from RT and confidence were highly correlated for each dataset (*p* < 0.05 for all datasets; mean *r* = 0.88), often approaching near-perfect agreement (Figure 9). Note that the correlation was somewhat reduced in Mazor_2025_Expt2, which involved relatively few trials per subject, as well as in Sherman_2016_JOCN_1 and Sherman_2016_JOCN_2, which employed an online staircase procedure that restricted *d*_*a*_ values within a limited range. Overall, these findings provide strong support for using RT-derived *d*_*a*_ to evaluate visual detection performance.

## Discussion

In visual detection tasks, observers often exhibit systematically different behaviors across trials involving stimulus presence versus absence.^6^^,^^18^^,^^30^^,^^31^ A prominent example is the asymmetry of type-1 ROC curves (Figure 1A), which, from an SDT perspective, indicates greater internal signal variance on stimulus-present trials compared to stimulus-absent trials.^6^^,^^13^ Capturing this asymmetry requires the unequal-variance SDT model (Figures 1A and 1B, solid navy lines), which relies on multiple data points in type-1 ROC space (i.e., multiple pairs of hits and FAs). Here, we evaluated the utility of RT data for fitting this model in comparison to the commonly adopted confidence-based approach.^13^

Estimates of the unequal-variance SDT parameters—the *SD* ratio (σ) and the mean difference (μ)—were highly consistent across the two methods (Figures 3, 4, 5, 7, and 8), though slightly lower when derived from RT than confidence (reductions of 6.3% and 8.6%, respectively). Consequently, both the numerator and denominator of the *d*_*a*_ index—a generalization of conventional *d′* for unequal-variance cases (see Equation 2)—were reduced to a similar extent, yielding close agreement between RT- and confidence-based estimates (mean *d*_*a*_ = 1.36 for RT and *1.43* for confidence; see Figures 3, 6, and 9). These results robustly demonstrate the utility of RT-based *d*_*a*_ estimation, while also suggesting that it should not be considered a full replacement for confidence-based analysis. Instead, RT-based *d*_*a*_ estimation is best regarded as a pragmatic solution in contexts where confidence ratings are not easily accessible.

Equally important, the conventional *d′*—derived solely from yes/no responses under the equal-variance SDT model (Figures 1A and 1B, dashed gray lines)—systematically overestimated detection performance relative to the *d*_*a*_ measures (Figures 3 and 6). Both *d*_*a*_ and *d′* represent the standardized mean difference between two internal distributions, scaled by the noise level.^13^ The present results indicate that failing to account for the structure of internal noise can lead to substantial misestimation of perceptual sensitivity.

Our results further showed that a major factor leading to this performance misestimation is the observer’s response bias. Type-1 ROCs in detection tasks typically show greater expansion on the left side (Figure 1). Consequently, even on the same unequal-variance ROC (i.e., iso-sensitivity curve in terms of *d*_*a*_), conventional *d′* calculated with more conservative (or lenient) criteria tends to overestimate (or underestimate) detection performance (see Figure 2).

Another relevant factor could be the amount of type-2 information carried by confidence or RT—the predictability of trial-by-trial response accuracy based on these variables.^12^ Greater type-2 information leads to a larger type-1 AUC, and, hence, higher *d*_*a*_.^31^ Here, the type-2 information conveyed by confidence or RT is often reported to be smaller than that predicted by the standard SDT model.^12^^,^^32^^,^^33^ This helps explain why *d*_*a*_, which incorporates these empirical data, tended to be smaller than *d′*, which is based solely on a single yes/no data point and presumes the type-2 information prescribed by the equal-variance SDT model. Therefore, our proposed RT-based unequal-variance SDT analysis provides a more precise sensitivity evaluation, grounded in more complete ROC data and effectively avoiding contamination from response bias.

One important implication of the present findings concerns comparisons between visual detection performance with other task paradigms, particularly pattern-discrimination tasks (e.g., “upward” vs. “downward” motion discrimination, or “target on the left” vs. “target on the right” spatial discrimination in a 2AFC design).^15^^,^^17^^,^^18^^,^^19^^,^^31^ Since type-1 ROC data in pattern-discrimination tasks tend to be more symmetrical, the use of conventional *d′* is less problematic in those contexts.^13^^,^^18^^,^^20^^,^^30^ Thus, applying *d′* uniformly to both detection and pattern-discrimination tasks may result in an inflated estimate of detection performance. Recognizing this concern, for example, a former study^34^ employed a costly base-rate manipulation to estimate *d*_*a*_ in a lesion study simulating blindsight behavior in macaque monkeys, which is commonly described as involving impaired detection despite preserved pattern discrimination.^35^^,^^36^^,^^37^^,^^38^^,^^39^ In contrast, utilizing the RT-derived *d*_*a*_ measure for both tasks would offer a low-cost approach to accurate performance assessment.

While much of the literature is interested in the parameter σ as a way to estimate *d*_*a*_, many studies have begun to focus on this parameter in its own right. For instance, individual differences in the σ parameter have been shown to account for variations in response bias.^40^ Moreover, both theoretical and empirical evidence converge on the finding that increased internal *SD* ratio in detection predicts enhanced metacognitive accuracy during pattern discrimination.^31^^,^^37^ In the present study, moderate correlations were found between the σ parameters estimated from RT and confidence data. This suggests that analyzing individual differences in internal variance using RT would be informative, particularly when a relatively large number of trials per subject is available.

A likely source of the unequal variance in detection tasks is the Poisson-like nature of neuronal firing, in which variance scales with the mean.^41^^,^^42^ Because the internal signal strength differs between target-present and target-absent trials, their variances naturally diverge as well. Notably, assuming that both the mean and variance of internal signals increase proportionally with stimulus intensity offers one account of Weber’s law—though a classic view instead assumes constant variance with the mean that scales logarithmically with stimulus intensity.^6^

Unequal variance is therefore relevant beyond yes/no detection tasks, including experiments that require stimulus classification along a single strength dimension. These tasks are collectively referred to as one-dimensional classification experiments and are often analyzed by fitting psychometric functions.^13^ In contrast to the pattern-discrimination tasks mentioned above, these could also be called “strength-discrimination” tasks. Incorporating RT-based estimates of internal variance in these designs may provide an opportunity to better capture the processes underlying the relevant behavior.

It is important to note that the present findings pertain specifically to the utility of RT for estimating *d*_*a*_, and should not be taken as evidence that RT can generally substitute for confidence in other analytical contexts. Indeed, our recent work on type-2 ROC analysis suggests that, while RT covaries with confidence through shared latent variables, it also exhibits unique properties that distinguish it from confidence.^32^ Future research is needed to further clarify the relationship between RT and confidence, as well as its underlying mechanisms.

Finally, although speculative, we would like to highlight several experimental factors that could potentially influence the utility of RT-based *d*_*a*_ estimation. For instance, dual-task designs that require switching between primary and secondary tasks may reduce RT precision for the primary task. Simultaneously recording a perceptual decision and an additional rating measure may also compromise RT precision; e.g., a yes/no response paired with a three-level rating involves six response keys, which could increase motor-related measurement noise. Moreover, excessively long response time limits may diminish subjects’ motivation to respond promptly. Further controlled studies will be essential for optimizing experimental designs that are best suited for RT-based SDT analysis.

In summary, SDT analysis based on RT provides a simple and practical solution, particularly as an alternative method for evaluating visual detection performance traditionally assessed through confidence-based approaches. This approach may also unlock deeper insights from existing datasets that lack confidence ratings or base-rate manipulations. Given its minimal implementation cost and capacity for rich behavioral assessment, RT-based ROC analyses hold great potential as a catalyst for advancing related research areas.

### Limitations of the study

The Supplementary Material extends the analysis to recognition memory datasets, motivated by the widespread use of the yes/no paradigm in this research domain (Figures S2–S10).^7^^,^^8^^,^^9^^,^^14^^,^^16^^,^^43^^,^^44^ In contrast to the findings for visual detection, the RT-based *d*_*a*_ in memory tasks tended to systematically underestimate task performance compared to confidence-based *d*_*a*_ and conventional *d′* (Figures S2, S5 and S9). As one source of this discrepancy, we found that RT in memory tasks carried much less information about response accuracy than confidence (Figure S10). Namely, the limited information contained in RT during memory tasks underlies the reduced value of RT-derived *d*_*a*_. One possible explanation is that, in memory tasks, prolonged retrieval processes may occasionally lead to correct responses, thereby weakening the expected relationship in which faster responses tend to indicate higher accuracy. Note that the studies analyzed here varied in their task procedures, such as the emphasis on response speed and the duration of response time limits. Thus, the observed dissociation should not be attributed solely to cognitive domain distinctions between perception and memory. Nonetheless, despite these inter-study differences, the results remained relatively consistent within each domain. Therefore, based on the current findings, we do not recommend applying RT-based *d*_*a*_ analyses to memory tasks. To reach more definitive conclusions, controlled experiments that directly compare perceptual and memory tasks are needed.

## Resource availability

### Lead contact

Requests for further information and resources should be directed to and will be fulfilled by the lead contact, Kiyofumi Miyoshi (miyoshi80@gmail.com).

### Materials availability

This study did not generate new unique materials.

### Data and code availability

• The original data used in this study are publicly available, with URLs provided in the key resources table.
• All code developed for this study has been deposited on GitHub: https://github.com/kiyomiyoshi/rt_type1_roc.
• Any additional information required to reanalyze the data reported in this article is available from the lead contact upon request.

## Acknowledgments

This work is supported by 10.13039/501100001691JSPS KAKENHI Grant Numbers 22K13870 and 25K00896, awarded to KM. HL is supported by the 10.13039/501100010446Institute for Basic Science, South Korea (Grant Number IBS-R015-D2). The funders have no role in study design, data collection and analysis, decision to publish, or preparation of the article.

## Author contributions

Conceptualization, K.M., D.R., and H.L.; methodology, K.M., D.R., and H.L.; investigation, K.M.; writing – original draft, K.M.; writing – review and editing, K.M., D.R., and H.L.; funding acquisition, K.M. and H.L.

## Declaration of interests

The authors declare no competing interests.

## STAR★Methods

### Key resources table

**Table:** 

REAGENT or RESOURCE	SOURCE	IDENTIFIER
**Deposited data**		

Raw data	OSF storage	https://osf.io/s46pr/overview
Raw data	OSF storage	https://osf.io/7v2d6/overview
Raw data	GitHub	https://github.com/ImagineRealityLab/METPRM

**Software and algorithms**		

R Project for Statistical Computing		RRID:SCR_001905
RStudio		RRID:SCR_000432
MATLAB		RRID:SCR_001622

### Experimental model and study participant details

This research is based solely on the reanalysis of existing data, and no additional samples were collected.

### Method details

#### Target datasets

We searched for yes/no visual detection datasets that met the following criteria.

(1) Both RT and confidence data are available.
(2) Confidence was measured using a scale with at least three levels.
(3) Separate, sequential responses were employed to record yes/no decisions and confidence ratings, since simultaneous reporting may compromise RT precision.
(4) The task did not involve a dual-task paradigm, which could introduce miscellaneous factors affecting RT, such as shifts in cognitive engagement.

We identified five studies that provided relevant data meeting our inclusion criteria.^2^^,^^3^^,^^18^^,^^28^^,^^29^ Data for three studies^18^^,^^28^^,^^29^ were obtained from the Confidence Database,^45^ while data for the remaining two studies^2^^,^^3^ were retrieved from their respective repositories. Each qualifying experimental condition was considered separately for the present analysis, resulting in a total of 11 datasets comprising 1,213 individuals (Table 1).

In three of these datasets—Mazor_2021_Expt7, Sherman_2016_JOCN_1, and Sherman_2016_JOCN_2—task difficulty was titrated within the condition using an online staircase procedure. This aspect may warrant consideration since aggregating across multiple difficulty levels could lead to elevated estimates of signal variance.

#### Data preprocessing

Since continuous confidence scales were employed in Dijkstra et al. (2024)^2^ and Mazor et al. (2021, 2025),^3^^,^^28^ we discretized these into three levels based on tertiles of each subject’s confidence data. Then, to ensure comparability with confidence-based analysis, for datasets with *n*-level confidence ratings, we discretized RTs into *n*-quantile bins per subject (Figure S1A).

For SDT analysis, we sorted each subject’s data into a 2 (stimulus class: stimulus present vs. stimulus absent) × 2*n* (yes/no responses × *n*-level confidence or RT) response frequency table. This procedure tabulates the hits and FAs associated with different confidence levels or RT bins (Figure S1B). To stabilize model fitting, we added a small constant (1/total number of trials) to each cell within the stimulus-present and stimulus-absent conditions.^12^^,^^46^

Lastly, we excluded individuals whose yes/no performance was below chance, resulting in the removal of 38 subjects (3.1%). Of those remaining, only cases in which the unequal-variance SDT model successfully converged using both RT and confidence data were retained, leading to the exclusion of 23 additional subjects (2.0%) and a final sample of 1,152 individuals.

#### Type-1 ROC

Trends in the aforementioned response frequency table can be visualized using type-1 ROC, which plots hit and FA rates across progressively more lenient criteria (Figure 1A). Specifically, the leftmost data point only considers yes responses with the highest confidence (or the fastest RT bin). The second point from the left includes yes responses with the highest and second-highest confidence levels (or the fastest and second-fastest RT bins), and so forth. This procedure produces 2*n* - 1 data points from *n* confidence levels (or RT bins), because including the most lenient “yes” responses (i.e., “no” responses with the highest confidence or fastest RT bin) maps to the point (1, 1) in type-1 ROC space (Figure S1C).

When constructed this way, the midpoint of type-1 ROC—the *n*-th point from the left in an *n*-level confidence (or RT bin) analysis—is determined solely by yes/no responses, representing the overall proportions of yes responses collapsed across all confidence levels (or RT bins). The positions of the remaining data points are influenced by confidence or RT. To the extent that these variables contribute to distinguishing stimulus classes (e.g., stimulus-present vs. stimulus-absent), type-1 ROC expands outward, increasing the AUC index. Thus, type-1 ROC visualizes how well primary perceptual judgment (e.g., yes/no response), in combination with an additional variable (such as RT or confidence), distinguishes between the states of external stimuli.

Note that “type-1 analysis” concerns performance in distinguishing objective features of the external world, defined independently of the observer. This is often contrasted with “type-2 analysis”, which evaluates how well a measurement (such as RT or confidence) distinguishes the observer’s own correct and incorrect responses.^10^^,^^11^^,^^12^ Type-2 performance—or the type-2 information contained in these measurements—can be assessed using type-2 ROC analysis or model-based metrics such as meta-*d′* (see Figure S10).

While empirical type-1 ROCs for pattern-discrimination tasks tend to be relatively symmetrical,^18^^,^^20^ those for detection tasks often exhibit marked asymmetry—characterized by a steep rise on the left and a shallower slope toward the right (Figure 1A). Importantly, from the unequal-variance SDT perspective, this asymmetry indicates greater variance for the target-present distribution compared to the target-absent distribution (Figure 1C).

#### Unequal-variance SDT analysis

We used maximum likelihood estimation to fit the unequal-variance SDT model to individuals’ data, separately considering RT and confidence. As described above, each subject’s data was arranged into a 2 × 2*n* response frequency table that crossed stimulus class with yes/no response and *n*-level confidence (or RT). Given values for μ, σ, and 2*n* – 1 response criteria, the unequal-variance SDT model specifies the predicted response probability for each cell. The likelihood of each cell was computed by raising its predicted probability to the observed frequency.^47^ We then took the logarithm of these cell-wise likelihoods and summed them up to obtain the model’s total log likelihood, which we numerically maximized using the optim() function in R.

In our implementation, the mean and *SD* of the target-absent distribution were fixed at 0 and 1, while the corresponding parameters of the target-present distribution (μ and σ) were estimated from the data (Figure 1C). These estimated parameters were then used to compute *d*_*a*_ according to Equation 2.

### Quantification and statistical analysis

All analyses were performed using the statistical software R (version 4.3.1). Paired *t*-tests were used to evaluate differences between conditions in Figures 4, 5, and 6. The boxplots in these figures depict the median, with the lower and upper hinges corresponding to the first and third quartiles. The whiskers extend to the smallest and largest values within 1.5 times the interquartile range (the distance between the first and third quartiles) from the hinges. Pearson’s correlation tests were conducted for the scatterplot data in Figures 7, 8, and 9. Sample sizes, defined as the number of subjects analyzed, are reported in Table 1.
