# Supplementary material for: Correcting for unequal variance in signal detection models using response time
Source: iScience. 2026 Feb 11;29(3):114998. doi: 10.1016/j.isci.2026.114998 (PMC12955650; doi:10.1016/j.isci.2026.114998)
Supplement: Document S1. Figures S1–S10 and Table S1 [file mmc1.pdf]

iScience, Volume *29*

Supplemental information

Correcting for unequal variance in signal detection models using response time

Kiyofumi Miyoshi, Dobromir Rahnev, and Hakwan Lau

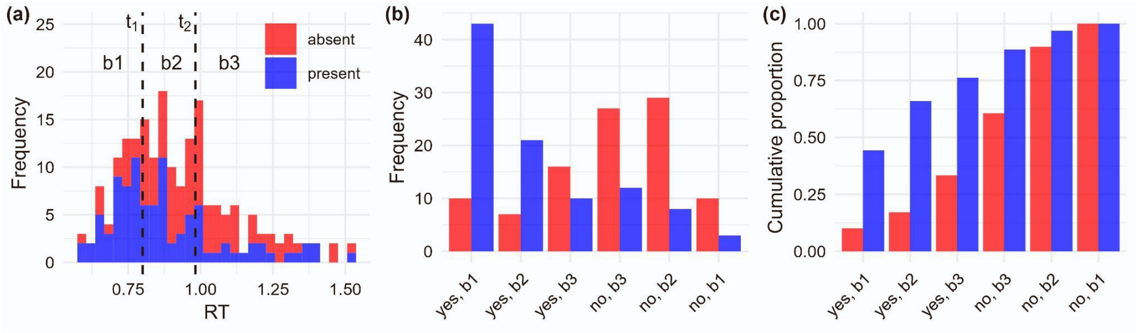

Figure S1. RT-based type-1 ROC construction. This figure illustrates our method for constructing type-1 ROC based on RT, using a three-level RT bin example. First, RTs are divided into three equal-sized bins, with stimulus class (absent/present) and response (yes/no) collapsed. Panel (**a**) illustrates this process, where t_1 and t_2 represent the cutoff thresholds defining the RT tertiles, and b1, b2, and b3 correspond to the fastest, second-fastest, and slowest RT bins, respectively. Trials for each stimulus class are thus characterized by an assigned response (yes/no) and an RT bin (three levels), classified in six response categories. Panel (**b**) shows the response frequency of these categories, arranged from left to right to indicate decreasing support for "yes" judgment (e.g., "no" responses in the fastest RT bin represent the weakest indication of "yes" judgment). In panel (**c**), cumulative response proportions are calculated sequentially from left to right for each stimulus class, which correspond to hit and FA rates in type-1 ROC space. Note that the cumulative proportions will always culminate at (1, 1), which is not considered a valid data point. Consequently, yes/no responses with n-level RT bins provide a total of 2n - 1 valid type-1 ROC data points.

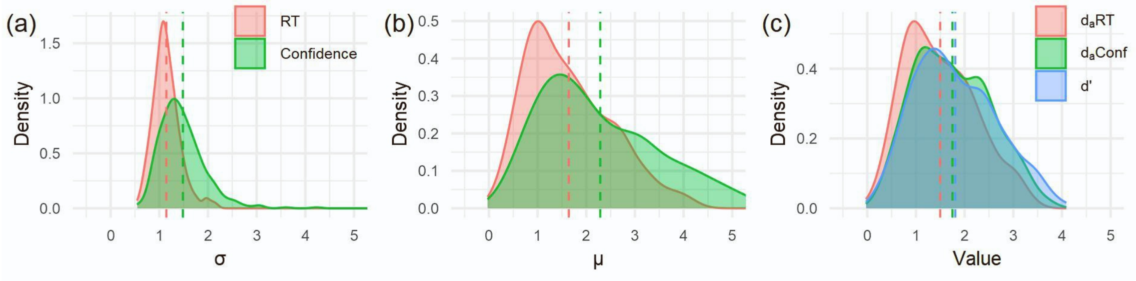

Figure S2. Estimated parameter distributions for recognition memory tasks. From the Confidence Database,[1] we selected five yes/no recognition memory studies that provided datasets meeting our inclusion criteria.[2–6] Each experimental condition from these studies was treated as a separate dataset, yielding a total of 10 datasets encompassing 449 individuals (see **Table S1** for details). One subject (0.2%) was excluded for below-chance yes/no performance. In addition, only those for whom the unequal-variance SDT model converged using both RT and confidence data were retained, resulting in the exclusion of 31 subjects (6.9%) and a final sample of 417 individuals. Here, data from these 417 individuals were aggregated for visualization (dashed lines indicate corresponding mean values). Unlike the results from the visual detection analysis, the d_a distributions derived from RT and confidence showed much discrepancy. Rather, RT-based d_a tended to underestimate task performance compared to confidence-based d_a or conventional d'.

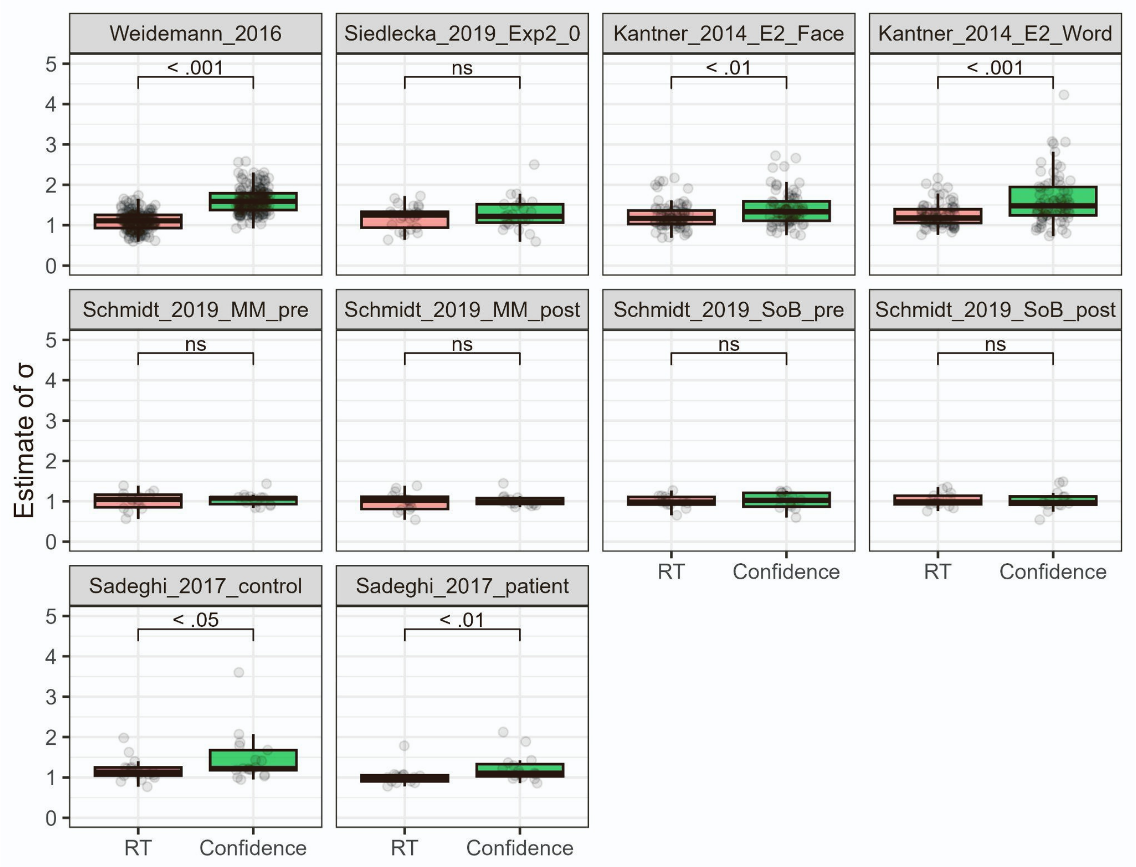

Figure S3. Estimates of σ for each recognition memory dataset. Overall, the σ values derived from RT tended to be smaller than those derived from confidence, with significant differences observed across the five datasets; the average of the dataset means of σ was 1.10 (*SD* = 0.1) for RT and 1.29 (*SD* = 0.27) for confidence, representing a 17% difference. This tendency was more pronounced than that observed in visual detection datasets. Statistical comparisons were conducted using paired *t*-tests.

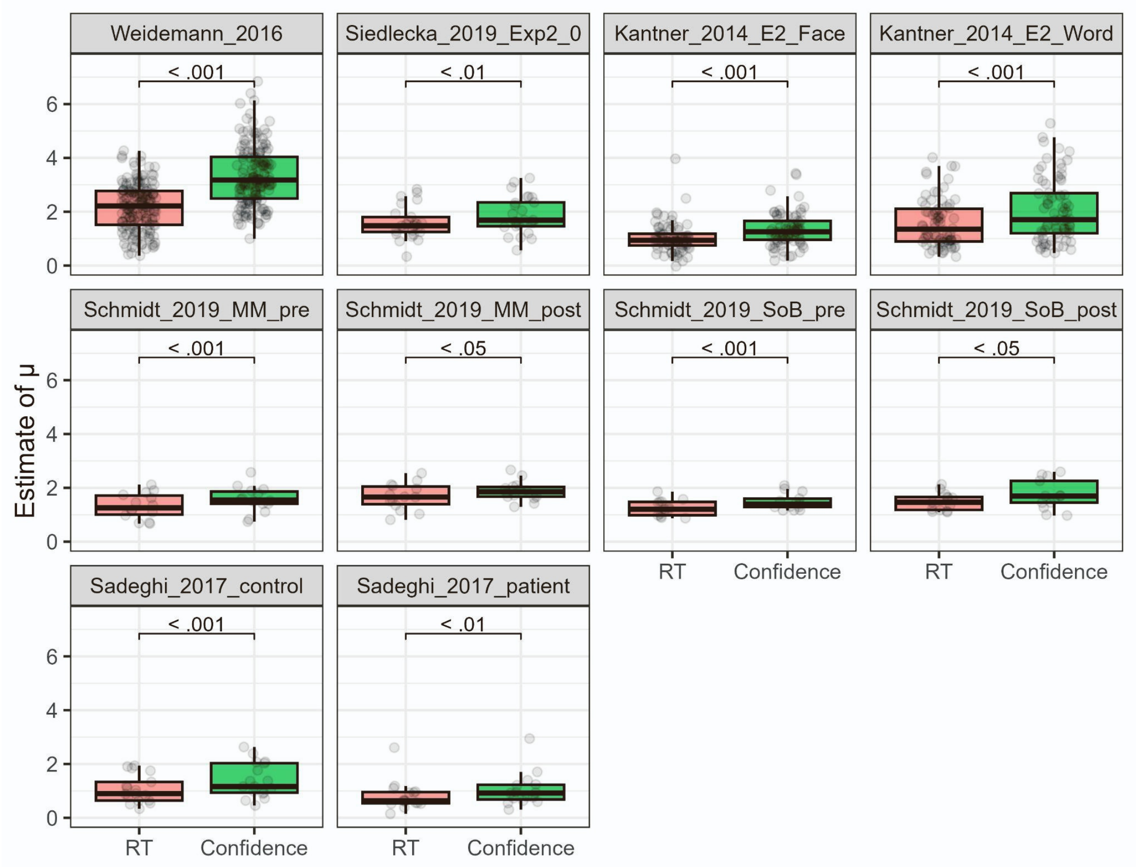

Figure S4. Estimates of μ for each recognition memory dataset. The μ values obtained from RT were generally smaller than those obtained from confidence, with significant differences observed across all datasets; the average of the dataset means of μ was 1.40 (*SD* = 0.39) for RT and 1.77 (*SD* = 0.63) for confidence, amounting to a 26% difference. This pattern was more marked than that seen in the visual detection datasets. Statistical comparisons were conducted using paired *t*-tests.

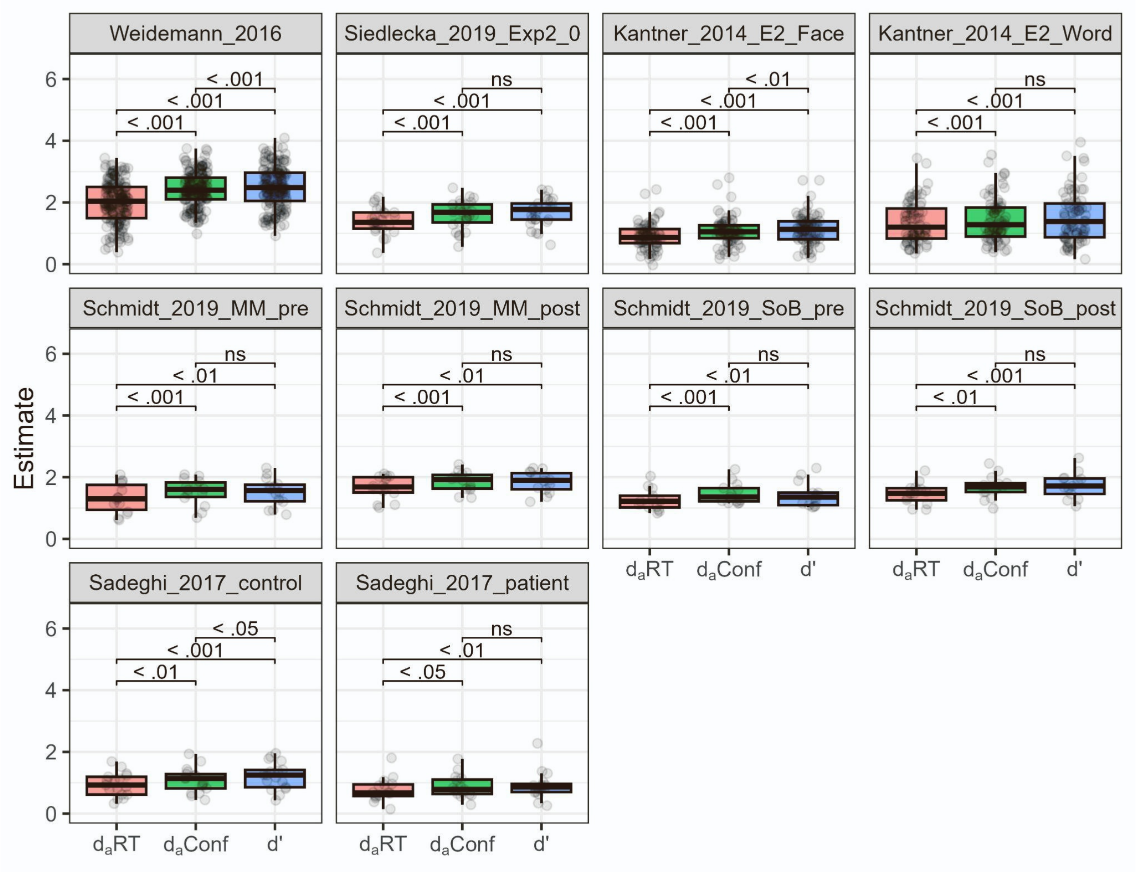

Figure S5. Sensitivity measures estimated for each recognition memory dataset. In contrast to visual detection, RT-based d_a tended to underestimate recognition memory performance compared to both confidence-based d_a and conventional d'. Across all datasets, RT-based d_a was significantly lower than d'. On the other hand, confidence-based d_a differed significantly from d' in only three datasets. The average of the dataset means of d' was 1.55 (*SD* = 0.44), which is 18% higher than that of RT-based d_a (*M* = 1.31, *SD* = 0.37) but only 3.3% higher than that of confidence-based d_a (*M* = 1.50, *SD* = 0.44). Paired *t*-tests were used for statistical comparisons (*p*-values uncorrected).

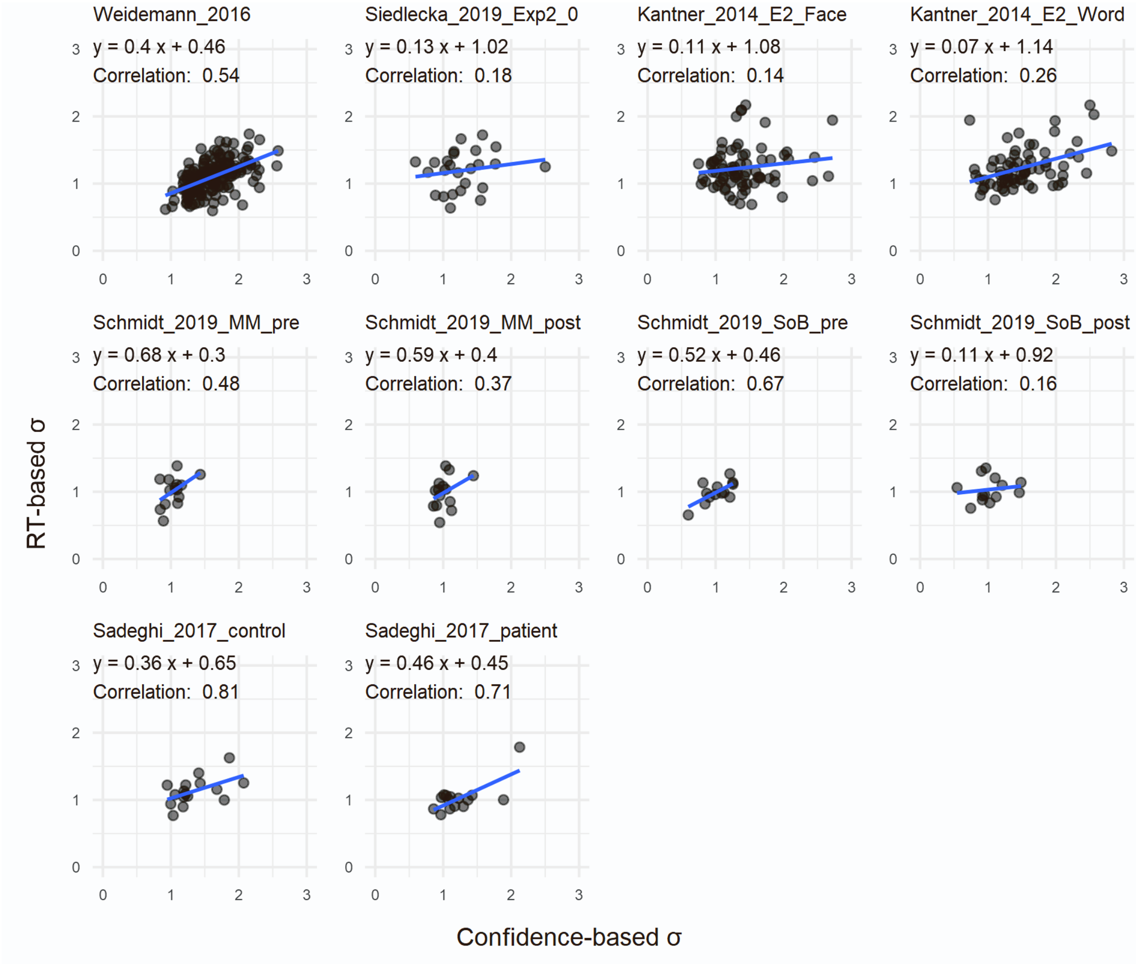

Figure S6. Correlation plots for the recognition memory σ parameters. Variability in correlation strength across the datasets is observed, presumably due to the sample size being relatively small.

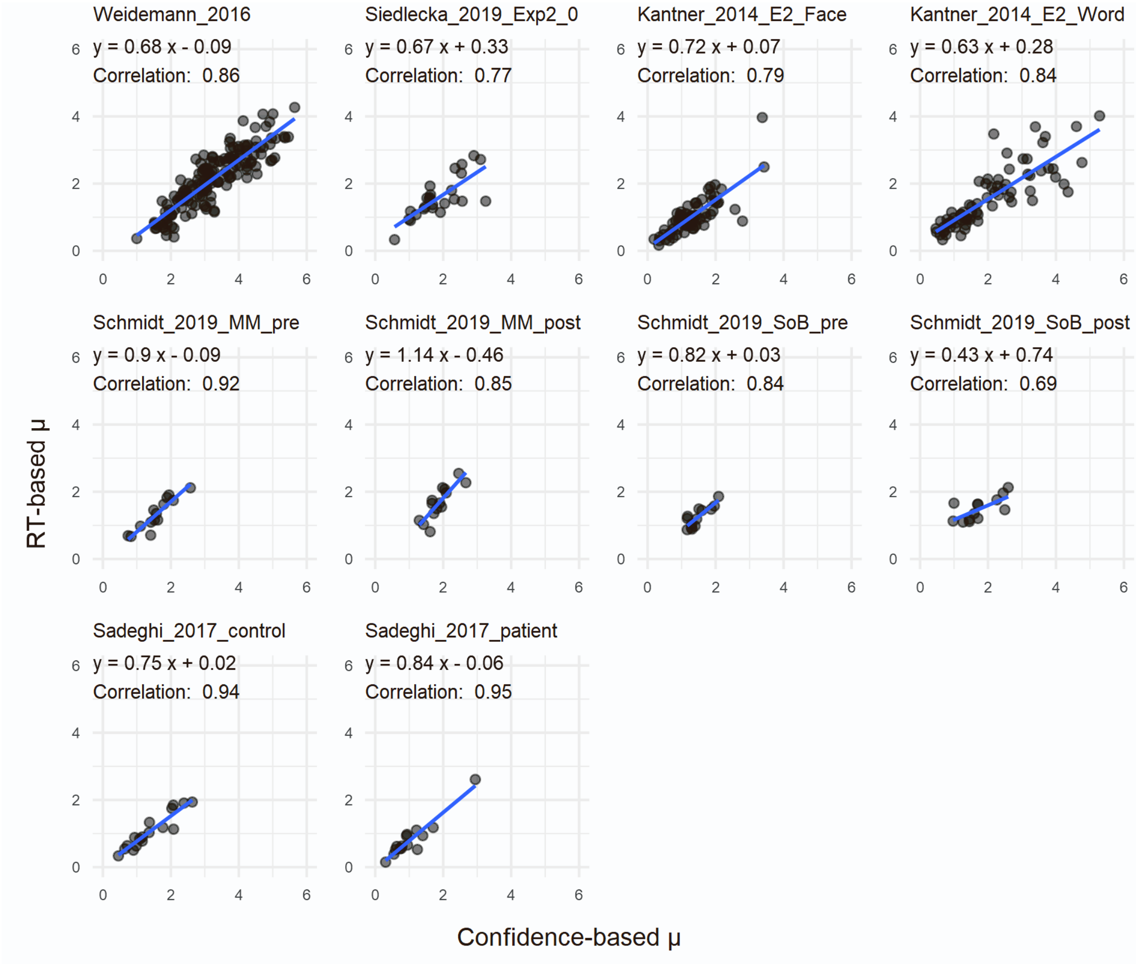

Figure S7. Correlation plots for the recognition memory μ parameters. Strong correlations were observed across all datasets as expected.

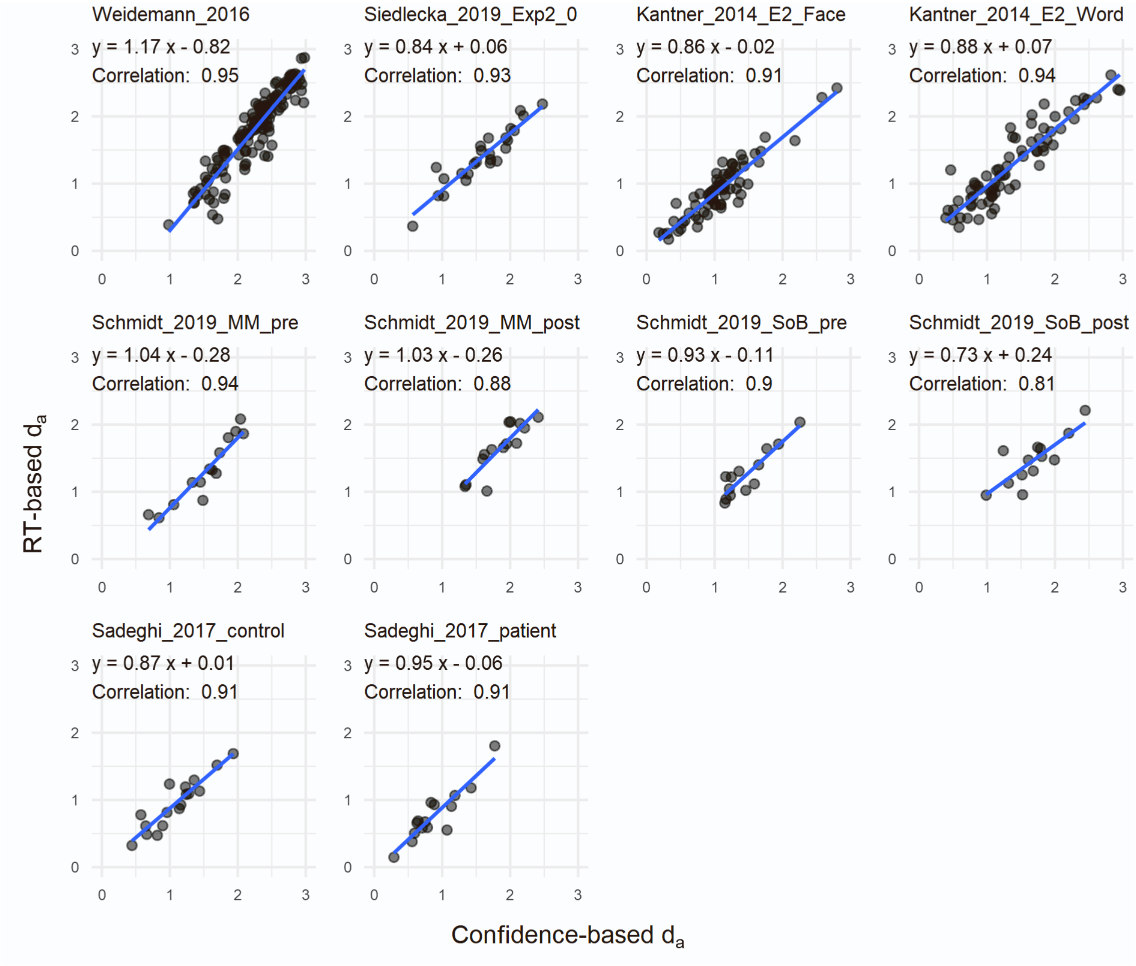

Figure S8. Correlation plots for recognition memory d_a. Estimates derived from RT and confidence demonstrated strong correlations, indicating that individual differences in d_a are maintained regardless of the variable used for estimation. However, caution is warranted since estimates based on RT tended to be smaller than those based on confidence (see **Figures S2** and **S5**).

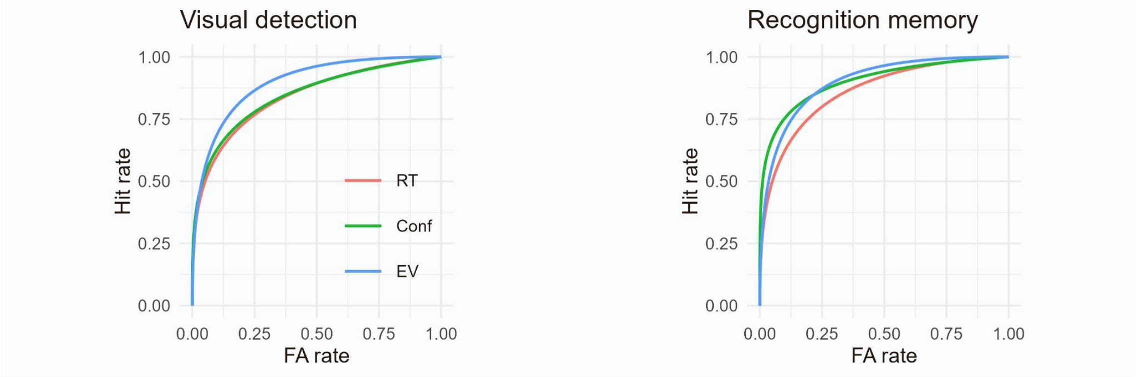

Figure S9. Model-based type-1 ROC curves. SDT predictions are derived from the average estimates across all individuals (*N* = 1,152 for visual detection and *N* = 417 for recognition memory). For visual detection, the curves based on RT and confidence substantially overlapped. Both demonstrated smaller AUCs than the blue curve, which represents the equal-variance model prediction derived solely from yes/no responses. In contrast, for recognition memory, the confidence-based curve showed markedly greater asymmetry and a larger AUC compared to the RT-based curve. This pronounced asymmetry—reflected in the steep rise at the left end of the ROC—is consistent with findings that highest-confidence yes responses in recognition memory tasks are strongly associated with hits and only rarely with FAs.[7,8] This pattern suggests that confidence ratings in recognition memory tasks possess high diagnosticity for distinguishing correct from incorrect responses, which likely contributes to the larger AUC and d_a values observed for the confidence-based analysis relative to the RT-based one.

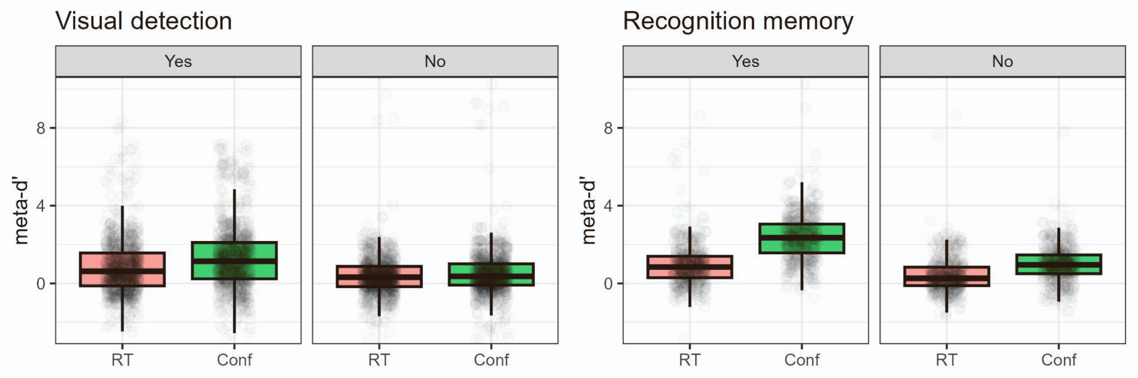

Figure S10. Response-specific meta-d′. The meta-SDT model[9] was fitted to all individual cases to examine the information that RT and confidence carry about response accuracy (type-2 information). The results were then aggregated for visualization (N = 1,152 for visual detection and N = 417 for recognition memory). Given that the present datasets indicate unequal variance in signal distributions, meta-d' was computed separately for yes and no responses.[10] For both yes and no responses, confidence carried more information about response accuracy than RT, particularly in recognition memory tasks. This disparity likely explains why d_a estimates based on confidence were generally larger than those based on RT in recognition memory datasets. This trend was notably less pronounced in visual detection tasks. Previous studies have also reported that confidence tends to be more informative about response accuracy in memory than in perceptual tasks.[11] These findings highlight the need for caution when applying the RT-based unequal-variance SDT analysis to recognition memory data.

Table S1. Recognition memory datasets

Dataset	Subjects	Trial/Sub	Confidence scale	Task	Specification
Weidemann_2016	159 (171)	2109-5866	5 levels	Word recognition	
Siedlecka_2019_Exp2_0	25 (26)	120	4 levels	Word recognition	*Condition* = 0
Kantner_2014_E2_Face	73 (74)	76	3 levels	Face recognition	*StimulusType* = "Face"
Kantner_2014_E2_Word	74 (74)	96	3 levels	Word recognition	*StimulusType* = "Word"
Schmidt_2019_MM_Pre	14 (14)	200	Continuous (1-6), made into 6 levels	Word recognition	*Condition* = "MM", Pre-training (trial 1 to 200)
Schmidt_2019_MM_Post	14 (14)	200	Continuous (1-6), made into 6 levels	Word recognition	*Condition* = "MM", Post-training (trial 201 to 400)
Schmidt_2019_SoB_Pre	13 (13)	200	Continuous (1-6), made into 6 levels	Word recognition	*Condition* = "SoB", Pre-training (trial 1 to 200)
Schmidt_2019_SoB_Post	13 (13)	200	Continuous (1-6), made into 6 levels	Word recognition	*Condition* = "SoB", Post-training (trial 201 to 400)
Sadeghi_2017_control	17 (25)	200	6 levels	Word recognition	*group* = "control"
Sadeghi_2017_patient	15 (25)	200	6 levels	Word recognition	*group* = "patient"

The “Subjects” column lists the number of individuals included in the analysis; values in parentheses reflect the counts prior to data exclusion. The “Trial/Sub” column refers to the number of trials per individual. The “Specification” column details which experimental conditions were selected from studies that included multiple conditions; variables shown in italics correspond to column headers in the source datasets.

Supplementary references

1. Rahnev, D., Desender, K., Lee, A.L.F., Adler, W.T., Aguilar-Lleyda, D., Akdoğan, B., Arbuzova, P., Atlas, L.Y., Balcı, F., Bang, J.W., et al. (2020). The Confidence Database. Nat. Hum. Behav. *4*, 317–325.

2. Kantner, J., and Lindsay, D.S. (2012). Response bias in recognition memory as a cognitive trait. Mem. Cognit. *40*, 1163–1177.

3. Sadeghi, S., Ekhtiari, H., Bahrami, B., and Ahmadabadi, M.N. (2017). Metacognitive deficiency in a perceptual but not a memory task in methadone maintenance patients. Sci. Rep. *7*, 7052.

4. Schmidt, C., Reyes, G., Barrientos, M., Langer, Á.I., and Sackur, J. (2019). Meditation focused on self-observation of the body impairs metacognitive efficiency. Conscious. Cogn. *70*, 116–125.

5. Siedlecka, M., Skóra, Z., Paulewicz, B., Fijałkowska, S., Timmermans, B., and Wierzchoń, M. (2019). Responses improve the accuracy of confidence judgements in memory tasks. J. Exp. Psychol. Learn. Mem. Cogn. *45*, 712–723.

6. Weidemann, C.T., and Kahana, M.J. (2016). Assessing recognition memory using confidence ratings and response times. R. Soc. Open Sci. *3*, 150670.

7. Yonelinas, A.P. (1994). Receiver-operating characteristics in recognition memory: Evidence for a dual-process model. J. Exp. Psychol. Learn. Mem. Cogn. *20*, 1341–1354.

8. Yonelinas, A.P. (1997). Recognition memory ROCs for item and associative information: the contribution of recollection and familiarity. Mem. Cognit. *25*, 747–763.

9. Maniscalco, B., and Lau, H. (2012). A signal detection theoretic approach for estimating metacognitive sensitivity from confidence ratings. Conscious. Cogn. *21*, 422–430.

10. Maniscalco, B., and Lau, H. (2014). Signal detection theory analysis of type 1 and type 2 data: Meta-d′, response-specific meta-d′, and the unequal variance SDT model. In The Cognitive Neuroscience of Metacognition (Springer Berlin Heidelberg), pp. 25–66.

11. Jin, S., Verhaeghen, P., and Rahnev, D. (2022). Across-subject correlation between confidence and accuracy: A meta-analysis of the Confidence Database. Psychon. Bull. Rev. *29*, 1405–1413.
